# Does Emotional Labor Matter for University Teaching? Examining the Antecedents and Consequences of University Teachers' Emotional Labor Strategies

**DOI:** 10.3389/fpsyg.2021.731099

**Published:** 2021-09-14

**Authors:** Jiying Han, Hongbiao Yin, Xin Yang, Feifei Wang

**Affiliations:** ^1^School of Foreign Languages and Literature, Shandong University, Jinan, China; ^2^Faculty of Education, The Chinese University of Hong Kong, Hong Kong, SAR China; ^3^School of Education, Northwest Normal University, Lanzhou, China; ^4^School of Translation Studies, Shandong University, Weihai, China

**Keywords:** emotional labor, emotional job demands of teaching, teaching support, teaching efficacy, university teachers

## Abstract

Following Grandey's integrative model of emotional labor and emotion regulation, this study examined the relationships between university teachers' reported use of various emotional labor strategies and some antecedents (i. e., perceived emotional job demands and teaching support) and teaching efficacy. A sample of 643 university teachers from 50 public higher education institutions in an East China province responded to a questionnaire survey. The data analysis based on descriptive statistics and structural equation modeling showed that surface acting impeded teaching efficacy in instructional strategy and learning assessment, while deep acting and expression of naturally felt emotions enhanced teaching efficacy in course design, instructional strategy, and learning assessment. For the antecedents of university teachers' emotional labor strategies, teachers perceived that the emotional job demands of teaching facilitated their use of surface and deep acting; in contrast, teachers' perceived teaching support decreased their use of surface acting and increased their use of expression of naturally felt emotions.

## Introduction

The concept of emotional labor was initially proposed to describe employees' management and display of emotions in the service sector (Hochschild, 1983). As schools have been perceived as complex arenas, it has been generally agreed that teaching is a form of emotional labor that meets all criteria for work that entails emotional labor (Yin and Lee, 2012). The emotional labor of teaching derives from teachers' interactions with students, colleagues, and parents (Yin et al., 2017), and teachers are expected to regulate their emotions and feelings according to the organizationally desired rules or guidelines (Wharton, 2009). As the emotional labor of teachers in primary and secondary schools has been widely examined (e.g., Yin et al., 2017; Zheng et al., 2018; Burić et al., 2020), a call for attention to the roles of emotion in the higher education teaching and learning process has been constantly noted (e.g., Thies and Kordts-Freudinger, 2019; Rinas et al., 2020). Some studies have preliminarily explored the complex emotions, such as anxiety, anger, love, and enjoyment of university teachers (e.g., Burić et al., 2020; Huang et al., 2020); however, how university teachers perceive and perform the emotional labor of teaching is still underexplored.

In higher education, emotion is necessary for successful teaching (Stupnisky et al., 2019a), and the increased accountability for the quality of teaching and learning in higher education has accentuated the need to focus on organizational behaviors of university teachers. Previous studies have preliminarily confirmed the coexistence of different emotional labor strategies among university teachers (e.g., Zhang and Zhu, 2008; Stupnisky et al., 2019b). Research concerning the relationship between university teachers' emotions and their teaching behaviors suggests that emotions of university teachers are related to university teachers' teaching practices and professional experiences (e.g., Postareff and Lindblom-Ylänne, 2011; Rinas et al., 2020). Mahoney et al.'s (2011) study with American professors indicated that, as professors seldom receive formal training in teaching, classroom management, and interpersonal activities, they are more likely to experience genuine positive emotional strategies in the long period of interactions with adult students. Considering the different emotional expressions between school and university teachers, little is known about the antecedents and consequences of emotions among university teachers (Thies and Kordts-Freudinger, 2019). Underpinned by Grandey's (2000) integrative model of emotional labor, this study explored the relationships between university teachers' reported use of various emotional labor strategies and their antecedents (i.e., perceived emotional job demands and teaching support) and teaching efficacy in a Chinese context.

## Literature

### Emotional Labor of Teaching

Emotional labor, a concept proposed in sociology to describe the nature of work in the service sector, was initially defined as “the management of feeling to create a publicly observable facial and bodily display” (Hochschild, 1983, p. 7). Early works (e.g., Ashforth and Humphrey, 1993; Morris and Feldman, 1996), stemming from different theoretical perspectives, could hardly reach an agreement on its definition and constructs (Bono and Vey, 2005; Grandey and Gabriel, 2015). Based on a review of those different conceptualizations of emotional labor, Grandey (2000) indicated the similarity in their underlying theme: individuals can regulate their emotional expressions at work. Therefore, emotional labor is defined as “the process of regulating both feelings and expressions for the organization goals in psychology” (Grandey, 2000, p. 97).

The past three decades have seen unprecedented growth in studies on emotional labor, and recent studies have examined emotional labor in a number of higher-level professional groups (Yin, 2015), such as lawyers and doctors (Wharton, 2009). According to Hochschild (1983), work that entails emotional labor requires face-to-face or voice-to-voice contact, the production of an emotional state in another person, and the exercise of a degree of control over emotional activities. As teaching has been acknowledged as work that fulfills those requirements in educational settings (Yin and Lee, 2012), a consensus has been reached that teaching is a form of emotional labor stemming from the interactions between teachers and students, and between teachers and their colleagues (Yin et al., 2017). With such acknowledgment, there has been a rapid expansion of research into teachers' emotional labor of teaching in the past two decades. However, as teachers' expression of emotions varies in different educational and cultural settings (Krone and Morgan, 2000), university teachers' emotional labor, which differs from that of school teachers, requires further inquiry.

### Grandey's Conceptual Model of Emotional Labor

Brotheridge and Grandey (2002) identified two fundamental approaches to conceptualizing emotional labor. The first one is job-focused emotional labor that denotes the level of emotional job demands such as the frequency of interactions and expectations to express certain emotions. The second one is employee-focused emotional labor denoting the process of emotional regulation and management such as the attempt to perform emotional labor and the use of different emotional labor strategies. With a particular focus on the management or modification of emotions in the workplace, Grandey (2000) proposed an integrative model for conceptualizing the mechanism of emotional labor in the workplace.

The integrative model suggests a linear process between emotional labor and its antecedents and consequences. Antecedents of emotional labor are situational cues, as well as individual and organizational factors. Situational antecedents include chronic expectations of interactions and acute events impacting on emotional labor. Individual antecedents are personal variables such as gender, expressivity, emotional intelligence, and affectivity. Organizational antecedents are factors contributing to the environment and situation in which emotional labor is performed, such as autonomy, and supervisor and coworker support. Consequences of emotional regulation are long-term consequences for employees (e.g., burnout and job satisfaction) and for organizations (e.g., performance and withdrawal behavior). Grandey's conceptual model integrates job-focused emotional labor (i.e., emotional demands of the job) and employee-focused emotional labor, which are, respectively, represented in terms of situational cues and emotional regulation. This model has served as a powerful framework with strong explanatory power in studies exploring the mechanism of emotional labor (Yin et al., 2017).

### Emotional Labor Strategies

Following general emotion theory, Hochschild (1983) proposed two widely recognized emotional labor strategies, both of which indicate deliberate management of emotions: surface acting through which one regulates emotional expressions, and deep acting in which one consciously modifies feelings to express the socially desired emotions. In line with this distinction, emotional regulation theory differentiates two types of emotion regulation strategies: response-focused and antecedent-focused emotion regulation (Gross, 1998). The former is concerned with modifying true feelings, whereas the latter is an approach where one modifies the emotion-inducing situation (Grandey, 2000). As both surface acting and deep acting imply that individuals should deliberately or consciously manage their own feelings, Ashforth and Humphrey (1993) further proposed the expression of naturally felt emotions, or genuine emotion, as a third strategy with a particular focus on expressed rather than felt emotions. The expression of naturally felt emotions refers to automatic emotion regulation which allows individuals to spontaneously experience and display the organizationally desired emotions (Zapf, 2002). This strategy was integrated into the dimensionality of emotional labor (Diefendorff et al., 2005), and subsequent studies supported the validation of the three emotional labor strategies (surface acting, deep acting, and the expression of naturally felt emotions) among school and university teachers (Zhang and Zhu, 2008; Yin, 2012).

Recently, in a qualitative study with Chinese school teachers (Yin, 2016), teachers' expression of naturally felt emotion was further divided into two categories: releasing and outpouring. The former refers to the genuine expression of positive emotions such as love and joy, and the latter denotes negative emotions like anger and sternness. It was also noted that China's high power distance and tradition of respecting teachers made outpouring negative emotions, which are often effective for teaching and maintaining discipline, acceptable in the Chinese context. However, this dichotomy of teachers' expression of naturally felt emotion remains largely untested in empirical studies.

### Emotional Job Demands of Teaching and Teaching Support as Antecedents of Emotional Labor

University teachers' emotions are triggered by various factors (Thies and Kordts-Freudinger, 2019). Emotional job demands are qualitative demands imposed by frequency, intensity, and variety of interpersonal interactions required by one's job (Brotheridge and Lee, 2002). Emotional job demands are usually considered stressful and detrimental, and therefore lead to unpleasant feelings, because meeting those demands may result in the depletion of resources and people value (Grandey, 2000). In educational settings, the emotional job demands of teaching emerge from teachers' intense and frequent interactions with students, colleagues, and administrators. These emotional job demands denote the emotional rules or display rules of teaching governing teachers' emotional expressions, such as showing positive emotions while suppressing negative ones (Yin and Lee, 2012). Therefore, in Grandey's (2000) integrative model, emotional job demands of teaching imply a situational variable in terms of interaction expectations that lead to the emotional labor.

Based on emotional regulation theory, Grandey's (2000) integrative model also suggests the significant relationships between organizational factors and emotional labor because the situation in which employees work would influence the level and type of emotional labor in which they engage. So far a very limited number of studies have explored the influence of organizational factors on teachers' emotional labor strategies (Yin et al., 2017). In this study, teaching support is used to denote organizational antecedents of teachers' emotional labor. Based on Chang et al. (2010) suggestion, university teachers' perceived teaching support is conceptualized in three dimensions: teaching resources which provide university teachers with favorable working conditions, peer support from colleagues that are helpful for achieving working goals, and administrative support from the organization that helps university teachers deal with the demands of their work.

### Teaching Efficacy as a Consequence of Emotional Labor

In line with previous findings that different emotional expressions would impact performance (Ashforth and Humphrey, 1993), Grandey's (2000) model postulates that emotional labor is related to a number of individual and organizational performances. Specifically, surface acting is negatively related to good performance, and deep acting would result in good performance (Gross, 1998). Teaching efficacy, which reflects teachers' judgments of their capability to positively influence the outcomes of teachers and students (Klassen and Tze, 2014), was proved to be an organizationally desired consequence of teachers' emotional labor (Yin et al., 2017). Empirical studies have generally agreed that efficacy beliefs (a sense of personal accomplishment) are positively related to deep acting and the expression of naturally felt emotion, but are negatively related to surface acting (e.g., Yin et al., 2017; Lee and Van Vlack, 2018; Zheng et al., 2018). However, it should be noted that a majority of the research on teachers' emotional labor has a particular focus on the effects of emotional labor on well-being indicators, whereas teachers' performance-based indicators such as teaching efficacy and effectiveness have been rarely addressed (Yin et al., 2017).

Unlike the abundant literature on teaching efficacy among school teachers, university teachers' efficacy has rarely been addressed (Postareff et al., 2008; Han et al., 2018). Chang et al. (2010) proposed to conceptualize faculty teaching efficacy as six dimensions, of which course design, instructional strategy, and classroom management are fundamental to classroom teaching efficacy. Adopting Chang's et al. (2010) framework, Han et al.'s (2018, 2020, 2021) recent empirical studies revealed several antecedents (e.g., teaching support, emotional job demands, stress) and outcomes (e.g., emotional exhaustion, engagement, teaching satisfaction) of university teachers' perceived efficacy. However, so far very little is known about the relationship between university teachers' emotional labor and teaching efficacy.

Based on the literature review, the following hypotheses were established.

**H1:** Teachers' perceived emotional job demands of university teaching are positively related to their reported use of surface acting (H1a) and deep acting (H1b), and negatively related to the expression of naturally felt emotion (H1c).
**H2:** Teachers' perceived teaching support is negatively related to their reported use of surface acting (H2a) and deep acting (H2b), and positively related to their expression of naturally felt emotion (H2c).
**H3:** Teachers' reported use of surface acting is negatively related to their perceived teaching efficacy in course design (H3a), instructional strategy (H3b), and learning assessment (H3c).
**H4:** Teachers' reported use of deep acting is positively related to their perceived teaching efficacy in course design (H4a), instructional strategy (H4b), and learning assessment (H4c).
**H5:** Teachers' reported use of expressions of naturally felt emotion is positively related to their perceived teaching efficacy in course design (H5a), instructional strategy (H5b), and learning assessment (H5c).

**Figure 1:**
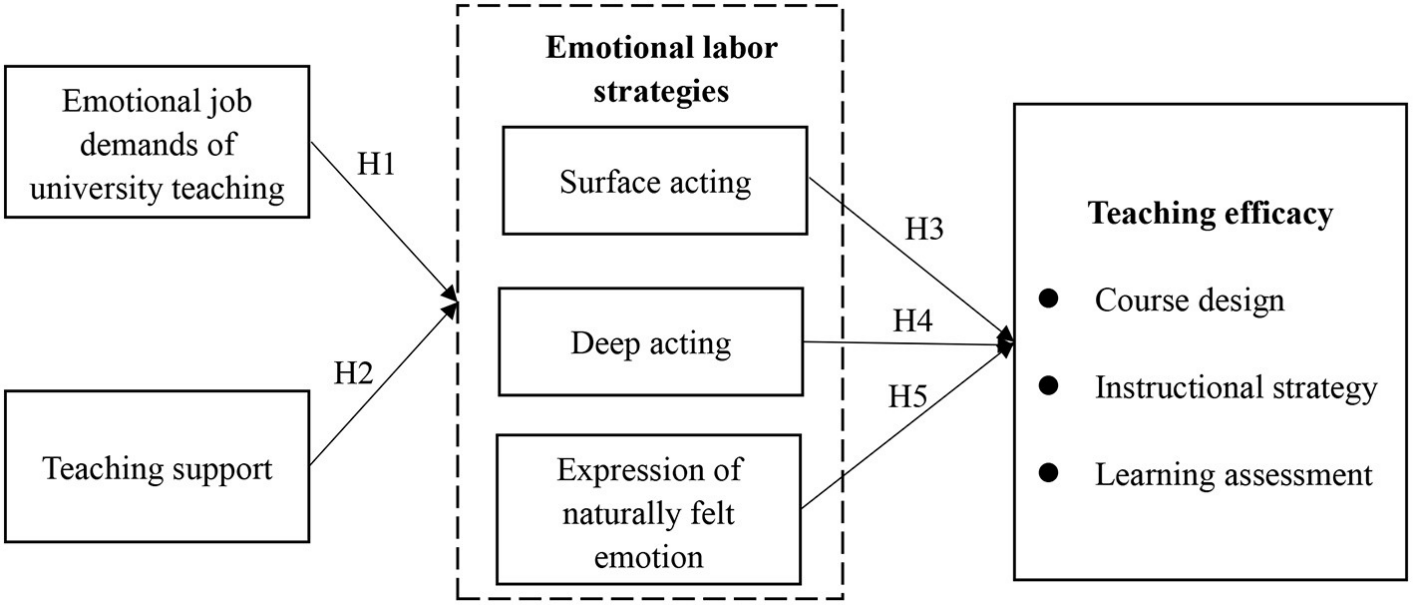
The hypothesized model.

Figure 1 shows the hypothesized model of this study.

## Method

### Participants

The sample consists of teachers from 50 public higher education institutions of Shandong province in East China. A total of 1,000 copies of the questionnaire were distributed to teachers during a university teacher training program which was initiated by the provincial Department of Education. All teachers were invited to voluntarily take part in the paper-based questionnaire survey. The analysis was based on usable responses from 65.4% (*N* = 643) of the invited sample, of which 398 (61.9%) were male and 245 (38.1%) were female. Regarding professional ranks, 51 (7.9%) were classified as teaching assistants (the beginning rank of HEIs in China), 149 (23.2%) as lecturers, 379 (58.9%) as associate professors, and 64 (10%) as professors. There were 256 (39.8%) teachers of liberal arts, 91 (14.2%) of science, 257 (40%) of technology, and 39 (6.1%) of medical science.

### Measures

The survey was conducted in December 2018. In addition to items related to participants' background information including gender, professional rank, and disciplinary distribution, four scales were included in the questionnaire. Appendix 1 provides details of the subscales and items.

### The Emotional Job Demands of the University Teaching Scale

The six-item scale was adapted from Yin's (2015) Emotional Job Demands of Teaching Scale. Specific steps included: (1) rephrasing item 1 [“I perform my teaching well, I have to spend most of my time interacting with others” (e.g., students and colleagues)] and 4 (“I have to use my emotions and behaviors to create a reassuring climate for my students”) by eliminating concerns of parents, and (2) adding two items (“In university teaching, I have to stimulate and elicit students' emotions so that they can devote themselves to learning” and “In university teaching, I have to manage my emotions and create an atmosphere which facilitates students' learning”). All items were scored using a 5-point Likert scale format ranging from 1 (strongly disagree) to 5 (strongly agree).

### The Revised Faculty-Perceived Teaching Support Scale

University teachers' perceived teaching support was assessed by Han et al.'s (2018) revised Faculty-Perceived Teaching Support Scale. It contains nine items in three subscales: teaching resources (e.g., “The university provides facilities and resources for teaching”), administrative support (e.g., “The administrators care about teachers' teaching effectiveness”), and peer support (e.g., “Colleagues share teaching experiences with and encourage me”). Each item is scored on a 4-point scale where higher scores suggest better perceived levels of teaching support.

### The Teacher Emotional Labor Strategies Scale

The 13-item scale (Yin, 2012) was adapted from the original Emotional Labor Strategies Scale (Diefendorff et al., 2005) with reference to teaching context. Slight changes were made by deleting the expressions of “parents” considering that university teachers are less likely to interact with parents. It is a scale assessing university teacher emotional labor strategies in three dimensions: surface acting (six items, e.g., “I just pretend to have the emotions I need to display for my job”), deep acting (four items, e.g., “I try to actually experience the emotions that I must show to students”), and expression of naturally felt emotions (four items, e.g., “The emotions I show students match what I spontaneously feel”). All items were rated on a 5-point Likert scale from 1 (strongly disagree) to 5 (strongly agree).

### The Faculty Teaching Efficacy Scale

Three subscales were selected from the original 28-item scale which was developed by Chang et al. (2011). They are course design (five items, e.g., “Have sufficient professional ability to teach the courses I am teaching”), instructional strategy (five items, e.g., “Teach according to students' various levels of readiness”), and classroom management (five items, e.g., “Maintain a good relationship with my students”). All items were scored on a 4-point scale, with higher scores indicating greater teaching efficacy.

### Data Analysis

The variances of participants' responses were checked during data screening, and cases were deleted if participants chose the same answer for all questions. Missing-value analysis using SPSS 23.0 revealed that <5% of the data were missing, and the expectation-maximization (EM) algorithm was used to deal with the missing data. The results of Skewness (ranging from |0.20| to |1.16|) and Kurtosis (from |0.02| to |1.78|) indicated that the sample distribution of this study approximated a normal distribution.

SPSS was used to conduct the descriptive statistics (mean and standard deviation) and correlations, and to examine the internal consistency (Cronbach's alpha). McDonald's omega was also computed using JASP to facilitate improved scale scrutiny. Mplus was used to conduct confirmatory factor analysis (CFA) and structural equation modeling (SEM) to test the hypothesized pathways. The regression model was constructed based on the assumption that correlations were allowed between the variables. The acceptance of the model was based on the following goodness-of-fit statistics: a Comparative Fit Index (CFI) and Tucker-Lewis Index (TLI) no <0.90, Standardized Root Mean Square Residual (SRMR) no more than 0.05, and a root mean square error of approximation (RMSEA) no more than 0.08 (Schreiber et al., 2006). Given the large sample size and considerable statistical power, all results were interpreted in the context of effect size using Gignac and Szodorai's (2016) guidelines for interpretation (small = 0.10 – <0.20, medium = 0.20 – <0.30, large ≥0.30).

## Results

### Construct Validity, Reliability, and Correlations

A series of CFA were conducted to test the factor structure of the scales. The Revised Faculty-Perceived Teaching Support Scale exhibited the strongest empirical and conceptual fit. The construct validity based on the second-order CFA indicated a good model fit (χ^2^ = 84.99, *df* = 24, *p* < 0.01, CFI = 0.98, TLI = 0.98, SRMR = 0.045, RMSEA = 0.063), with factor loadings ranging from 0.53 to 0.94.

Ensuring the construct validity of the Emotional Job Demands of University Teaching Scale was rather complicated due to the lower factor loading of item 1 (<0.40; “To teach well, I have to be considerate and think from the point of view of my students and colleagues”). Factor loadings of the remaining five items ranged from 0.47 to 0.82.

The CFA fit indices of both the Teacher Emotional Labor Strategies Scale (χ^2^ = 387.95, *df* = 74, *p* < 0.01, CFI = 0.94, TLI = 0.93, SRMR = 0.044, RMSEA = 0.051) and the Faculty Teaching Efficacy Scale (χ^2^ = 664.91, *df* = 87, *p* < 0.01, CFI = 0.93, TLI = 0.91, SRMR = 0.045, RMSEA = 0.062) were within acceptable limits. Factor loadings of the two scales ranged from 0.62 to 0.88 and 0.70 to 0.87, respectively.

Table 1 shows the descriptive statistics of all factors, reliability, and correlation coefficients between latent factors. The internal consistency of all measures was within acceptable limits. The correlation matrix shown in Table 1 indicates that all constructs were significantly related with each other apart from the relationship between emotional job demands and expression of naturally felt emotions and that between deep acting and course design. However, the association of teaching satisfaction with deep acting, and that of deep acting with instructional strategy and classroom management were very small (|*r*| < 0.10) and so we caution against making substantive interpretations.

**Table 1:** Correlations, descriptive statistics and internal consistency estimates of all standardized measures (*N* = 643).

	**EJD**	**TS**	**SA**	**DA**	**NF**	**CD**	**IS**	**CM**
Emotional job demands (EJD)	–							
Teaching support (TS)	0.07	–						
Surface acting (SA)	0.12^**^	−0.14^**^	–					
Deep acting (DA)	0.27^**^	0.09^*^	0.36^**^	–				
Expression of naturally felt emotions (ENFE)	0.05	0.20^**^	−0.54^**^	−0.16^**^	–			
Course design (CD)	0.21^**^	0.24^**^	−0.16^**^	0.05	0.23^**^	–		
Instructional strategy (IS)	0.27^**^	0.32^**^	−0.19^**^	0.08^*^	0.26^**^	0.76^**^	–	
Classroom management (CM)	0.26^**^	0.37^**^	-0.19^**^	0.09^*^	0.27^**^	0.74^**^	0.81^**^	–
Cronbach's alpha	0.78	0.90	0.86	0.86	0.92	0.89	0.92	0.89
McDonald's omega	0.78	0.89	0.86	0.86	0.92	0.89	0.92	0.90
Mean	4.16	4.13	2.87	3.59	3.68	4.85	4.62	4.74
Standard deviation	0.47	0.88	0.75	0.64	0.69	0.71	0.78	0.70

* *p < 0.05*,

** *p < 0.01 (two-tailed); Factors in bold are statistically significant (p < 0.05) and have at least a small effect size (r > –|0.10|)*.

### Structural Equation Model

**Figure 2:**
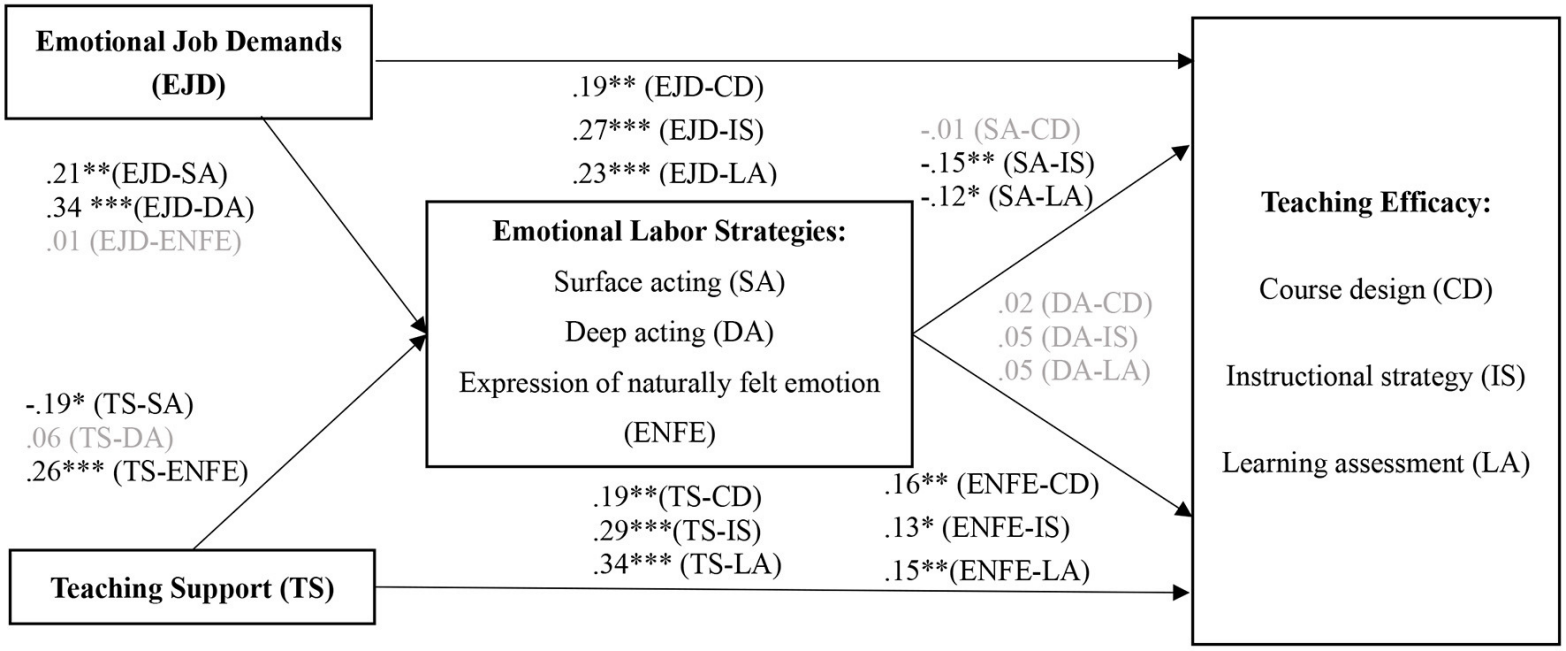
Effects of emotional job demands and teaching support on university teachers' teaching efficacy via emotional labor strategies (*N* = 643). *p < 0.05, **p < 0.01, ***p < 0.001; Goodness-of-fit indices: χ^2^ = 2423.54, *df* = 831, *p* < 0.01, CFI = 0.91, TLI = 0.91, SRMR = 0.08, RMSEA = 0.055.

SEM analysis was conducted to examine the effects of emotional job demands and teaching support on university teachers' teaching efficacy through various emotional labor strategies. The SEM results exhibited an acceptable fit with the data (χ^2^ = 2423.54, *df* = 831, *p* < 0.01, CFI = 0.91, TLI = 0.91, SRMR = 0.080, RMSEA = 0.055). Figure 2 shows the details of the SEM analysis.

The results suggested that teachers' perceived emotional job demands of teaching were positively related to their reported use of surface acting (β = 0.21, *p* < 0.01) and deep acting (β = 0.34, *p* < 0.001) with a medium to large effect size, but were not significantly related to expression of naturally felt emotions. Thus, H1a and H1b were supported, and H1c was rejected. Teachers' perceived teaching support was negatively related to their reported surface acting (β = −0.19, *p* < 0.05) with a small effect size, positively related to expression of naturally felt emotions (β = 0.26, *p* < 0.001) with a medium effect size, and not significantly related to deep acting. Thus, H2a and H2c were supported and H2b was rejected. Teachers' reported use of surface acting was negatively related to their perceived teaching efficacy in instructional strategy (β = −0.15, *p* < 0.01) and learning assessment (β = −0.12, *p* < 0.05) with a small effect size, but was not significantly related to course design. Thus, H3b and H3c were supported, whereas H3a was rejected. In addition, it was found that deep acting did not have any significant effect on university teachers' teaching efficacy. Thus, H4 was rejected. In contrast, expression of naturally felt emotion exhibited positive associations with teaching efficacy for course design (β = 0.16, *p* < 0.01), instructional strategy (β = 0.13, *p* < 0.05), and learning assessment (β = 0.15, *p* < 0.01), supporting H5.

### Mediation Analysis

A bootstrapping test based on 5,000 samples was conducted to examine the significance of the mediating effects. The results of the mediation analysis are presented in Table 2. In short, the results revealed that both teachers' perceived emotional job demand and teaching support had positive and significant effects on their teaching efficacy beliefs. In terms of the mediating effects, it was found that surface acting significantly mediated the effects of emotional job demands on teachers' efficacy for instructional strategy. Meanwhile, expression of naturally felt emotion significantly mediated the effects of teaching support on teachers' three types of efficacy beliefs, namely, the efficacy for course design, instructional strategy, and learning assessment. In addition, the results also showed that deep acting significantly mediated the relationships between teaching support and teachers' efficacy for instructional strategy and learning assessment. However, as the effect size of these statistically significant indirect effects was very close to zero, the mediating effects may not have enough practical meaning.

**Table 2:** The estimates of direct effects and indirect effects of the 95% confidence intervals.

**Dependent variable**	**Independent variable**	**Mediator**	**Direct effect**	**Indirect effect**	**95% CIs**		***R* ^**2**^**
					**Lower 2.5%**	**Upper 2.5%**	
Course design	Emotional job demands		0.19^**^	−0.01	−0.05	0.03	0.13
		Surface acting		−0.02	−0.04	0.01	
		Deep acting		0.01	−0.03	0.04	
		Expression of naturally felt emotion		0.00	−0.02	0.02	
	Teaching support		0.27^***^	−0.01	0.02	0.10	
		Surface acting		−0.03	−0.01	0.04	
		Deep acting		0.02	−0.01	0.01	
		Expression of naturally felt emotion		**0.00**	0.01	0.08	
Instructional strategy	Emotional job demands		0.19^**^	0.06	−0.06	0.03	0.24
		Surface acting		**0.02**	−0.06	−0.01	
		Deep acting		0.00	−0.02	0.05	
		Expression of naturally felt emotion		0.04	−0.02	0.02	
	Teaching support		0.29^**^	0.07	0.03	0.11	
		Surface acting		**0.01**	0.01	0.06	
		Deep acting		–**0.01**	0.00	0.01	
		Expression of naturally felt emotion		**0.00**	0.01	0.07	
Learning assessment	Emotional job demands		0.23^***^	−0.01	−0.05	0.04	0.25
		Surface acting		−0.02	−0.05	0.00	
		Deep acting		0.01	−0.02	0.05	
		Expression of naturally felt emotion		0.00	−0.02	0.02	
	Teaching support		0.34^***^	0.06	0.02	0.10	
		Surface acting		0.02	−0.01	0.05	
		Deep acting		**0.00**	0.01	0.07	
		Expression of naturally felt emotion		**0.04**	0.02	0.08	

*Bootstrap samples = 5,000*;

** *p < 0.01*,

*** *p < 0.001. Values in bold are significant mediation effect*.

## Discussion

This study addresses the paucity of research on faculty emotions (Mahoney et al., 2011; Thies and Kordts-Freudinger, 2019) and adds literature to the research of university teachers' emotional labor. The study provides empirical evidence of the application of Grandey's (2000) integrative model of emotional labor in the context of higher education, and it helps to verify the linear process between situational antecedents of emotional job demands, the organizational factor of teaching support, university teachers' reported use of emotional labor strategies, and organizational desired consequence of teaching efficacy. The results indicated that the expression of naturally felt emotion was university teachers' commonly used emotional labor strategy, and it typically resulted from their perceived teaching support rather than from their emotional job demands. The results revealed the negative influence of surface acting and the positive influence of expression of naturally felt emotion on teaching efficacy. The results of mediation analysis also supported the significant mediating roles of surface acting and expression of naturally felt emotion between the effects of emotional job demand and teaching support on university teachers' teaching efficacy. In general, findings of this study highlight the significance of emotional labor for university teachers, and give credence to Grandey's proposed process of teachers' emotional labor regulation.

### University Teachers' Reported Use of Emotional Labor Strategies and Its Antecedents

Results of this study revealed the relationships between university teachers' reported use of emotional labor strategies and its antecedents of their perceived emotional job demands and teaching support. First, our results were consistent with findings of previous studies with school teachers (e.g., Näring et al., 2012; Yin et al., 2017) in that teachers' perceived emotional job demands were positively related to their reported use of surface acting and deep acting. This indicates that when university teachers meet emotional demands through interactions with students and colleagues, they are more likely to be pressured to manage their emotions. As Mahoney et al. (2011) noted, university teachers may be more pressured to manage their emotions than school teachers because ratings from their adult students constitute a key factor in teachers' performance appraisal.

However, unlike the established negative relationship between school teachers' reported emotional job demands and expression of naturally felt emotions (Näring et al., 2012; Yin, 2015; Yin et al., 2017), our study did not reveal a significant relationship between these two variables among university teachers. Meanwhile, our study found that university teachers reported a higher level of expression of naturally felt emotion than surface and deep acting, indicating that they are more likely to engage in genuine expression of emotion. The significance of being genuine for university teachers echoed Mahoney et al.'s (2011) study with American professors which believed that university teachers are more likely to perceive being genuine as the best way for several reasons. On one hand, university teachers typically focused more on the cognitive part of the job, and they would be uncomfortable about managing their emotions. On the other hand, they may be less skilled in employing emotional regulation strategies, because they have less formal training in teaching and emotion display rules.

Being the most commonly used emotional labor strategy, university teachers' expression of naturally felt emotion typically resulted from their perceived teaching support rather than from their emotional job demands. This finding indicates that university teachers' perceived support of teaching is an important job resource that makes them feel free to express their genuine emotions. Meanwhile, the negative relationship between university teachers' perceived teaching support and their reported use of surface acting indicates that a supportive teaching environment helps reduce their efforts to suppress or disguise their genuine emotions. In addition, our results revealed no significant relationship between teaching support and deep acting. Unlike surface acting which is concerned with teachers' modification and suppression of true feelings, deep acting stresses the process of cognitive modification of internal feelings to comply with the required emotional expression (Grandey, 2000). Yin et al. (2017) suggested that deep acting might be independent of the work environment as it is more likely to be related to teachers' philosophy and personal skills. This might help explain the non-significant relationship between teaching support and deep acting.

### University Teachers' Reported Use of Emotional Labor Strategies and Its Consequences

Unlike previous studies of teachers' emotional labor that have frequently focused on consequences of psychological well-being such as emotional exhaustion and job satisfaction (e.g., Mahoney et al., 2011; Keller et al., 2014), this study revealed the relationship of teachers' emotional labor with the organizational consequences of teaching efficacy. Bandura (1997) claimed that individuals rely partly on information conveyed by emotional states when judging their own capabilities. It also gives credence to the relationships between emotional labor strategies and teaching efficacy. Generally, our findings revealed some negative effects of university teachers' reported use of surface acting, positive effects of expression of naturally felt emotion, and non-significant effects of deep acting. These findings support the majority of the hypotheses on the relationships between university teachers' reported use of emotional labor strategies and their teaching efficacy beliefs, except for H3a and H4.

The positive influence of university teachers' reported use of expression of naturally felt emotion on teaching efficacy was consistent with Yin et al.'s (2017) study with school teachers. These results indicate that genuine expression of emotions helps university teachers make positive appraisals of their teaching capability. Specifically, they would be more efficacious in their abilities of course design, instructional strategy use, and learning assessment. However, the negative relationship between university teachers' reported surface acting and teaching efficacy, especially in instructional strategy and learning assessment, confirms the harmful effect of surface acting as expected. These findings were consistent with the claim that emotional labor has both positive and negative effects depending on the salient strategy (Zhang and Zhu, 2008). Accordingly, university teachers' emotional labor tends to have detrimental effects when surface acting prevails, and to have favorable effects when genuine expression is prevalently used.

Unlike our hypotheses, the results of this study found that deep acting had no significant effects on university teachers' teaching efficacy. This finding reminds us of the different nature of deep acting compared with surface acting and expression of naturally felt emotion. As stated earlier, deep acting put more emphasis on the internal cognitive modification of feelings, but both surface acting and expression of naturally felt emotion stress the expressive emotions, gestures, or behaviors (Grandey, 2000; Yin et al., 2017). At the same time, as a desirable organizational outcome, teaching efficacy, unlike those personal outcomes such as well-being indicators, emphasizes teachers' confidence in their abilities to accomplish specific teaching tasks or behaviors. Moreover, deep acting, which requires teachers to change their feelings and display required emotional expressions, is more likely to lead to depletion of resources and energy. Therefore, Zhang and Zhu's (2008) study with Chinese university teachers reported that, compared with authenticity, deep acting was a more effective predictor of burnout. These reasons may explain why deep acting was not significantly related to teaching efficacy in this present study.

Although both surface acting and naturally felt emotion were significantly related to teaching efficacy, their effects differed in magnitude. Our results reveal that university teachers' expression of naturally felt emotion had relatively stronger associations with teaching efficacy in the expected direction. Based on these results, we can conclude that expression of naturally felt emotion is beneficial in terms of bringing desirable organizational consequences in higher education.

### Limitations and Suggestions for Future Research

This study reveals some significant findings on the relationships between university teachers' emotional labor strategies and some antecedents (i.e., perceived emotional job demands and teaching support) and consequence (teaching efficacy). Three limitations need to be acknowledged, which can stimulate future research. First, the current study is limited by the cross-sectional research design which is insufficient to confirm the causal relationships between the variables. Further studies may consider a longitudinal design to determine the directionality of the regression paths. Second, although the current study was based on a sample of university teachers from different higher education institutions, evidence from Shandong province alone may limit the generalizability of the findings. Considering the large territory of mainland China, future research is expected to better understand the relationships between these variables with a more representative sample. Third, this study focused primarily on situational and organizational antecedents on university teachers' emotional labor strategies. As individual factors were also identified to affect emotional labor in Grandey's model, future research may further take individual antecedents as covariates.

## Implications

This study establishes a quantitative framework for detecting Grandey's (2000) integrative model of emotional labor among university teachers in China. Clarifications of the relationships between university teachers' emotional labor strategies and some antecedents and consequences provide both university teachers and administrators with new understandings of the significance of teachers' emotional labor. Although this study targeted university teachers from mainland China, several practical applications of our findings can be a reference for scholars and practitioners from other countries who may have an interest in teacher emotion research.

First, the results of this study have significant implications for the understanding of how university teachers' perceived climate plays a role in their adoption of emotional labor strategies. Specifically, considering different roles of university teachers' perceived emotional job demands and teaching support, a favorable climate could be created to make university teachers feel more supportive and free to express their genuine emotions, which would help boost their confidence in teaching practice. Meanwhile, in view of the positive relationship between emotional job demands and teachers' use of both surface and deep acting strategies, university teachers should be encouraged to use cognitive techniques to change their feelings rather than express unfelt feelings in the face of emotional job demands.

Second, this study revealed the positive effects of university teachers' use of genuine expression of emotions, and negative effects of surface acting, on teaching efficacy. This reminds us of the significance of university teachers carefully choosing their emotional labor strategies in practice. Some faculty development programs are needed to improve teachers' understanding of the roles of emotional labor in their work and the different natures of emotional labor strategies. Specifically, faculty members should feel free to express their genuine emotions and reduce their use of surface acting strategies to effectively improve their teaching efficacy.

## Data Availability Statement

The raw data supporting the conclusions of this article will be made available by the authors, without undue reservation.

## Ethics Statement

The studies involving human participants were reviewed and approved by the Survey and Behavioral Research Ethics Committee at the Chinese University of Hong Kong. The patients/participants provided their written informed consent to participate in this study.

## Author Contributions

JH and XY collected and analyzed the data and wrote the first draft of the manuscript. HY designed the research and wrote the first draft of the manuscript. FW helped with data collection and finalized the manuscript. All authors contributed to the article and approved the submitted version.

## Funding

This work was supported by Young Scholars Programme of Shandong University under Grant number 2017WLJH09.

## Conflict of Interest

The authors declare that the research was conducted in the absence of any commercial or financial relationships that could be construed as a potential conflict of interest.

## Publisher's Note

All claims expressed in this article are solely those of the authors and do not necessarily represent those of their affiliated organizations, or those of the publisher, the editors and the reviewers. Any product that may be evaluated in this article, or claim that may be made by its manufacturer, is not guaranteed or endorsed by the publisher.
