# Supplementary material for: Does Emotional Labor Matter for University Teaching? Examining the Antecedents and Consequences of University Teachers' Emotional Labor Strategies
Source: Front Psychol. 2021 Sep 14;12:731099. doi: 10.3389/fpsyg.2021.731099 (PMC8476905; doi:10.3389/fpsyg.2021.731099)
Supplement: Supplementary file 1 [file Data_Sheet_1.docx]

# Appendix：The scales used in the study

***Emotional Job Demands for University Teaching***

1. To teach well, I have to be considerate and think from the view of point of my students and colleagues.

2. To teach well, I have to spend a lot of time on every student whom I taught.

3. I have to use my emotions and behaviors to create a reassuring climate for my students

4. In university teaching, I have to stimulate and elicit students’ emotions so that they can devote themselves to learning

5. In university teaching, I have to manage my emotions and create an atmosphere facilitating students’ learning.

***The Revised Faculty-Perceived Teaching Support Scale***

*Teaching resources*

1. The university provides the facilities and resources for teaching.

2. The university provides the technology and software resources for teaching.

3. The university provides the facilities and resources to help you improve student learning.

*Administrative support*

1. The administrators care about teachers’ teaching effectiveness.

2. The administrators have a comprehensive mechanism that rewards quality teaching.

3. The administrators require high teaching quality from teachers.

*Peer support*

1. The colleagues provide teaching demonstration opportunities for me to observe other

colleagues’ teaching.

2. The colleagues encourage and support me if I have an experiment with my teaching.

3. The colleagues share teaching experiences with me.

***Teacher Emotional Labor Strategy Scale***

*Surface acting*

1. I put on a ‘show’ or ‘performance’ when interacting with students or their parents.

2. I show feelings to students or their parents that are different from what I feel inside.

3. I fake the emotions I show when dealing with students or their parents.

4. I just pretend to have the emotions I need to display for my job.

5. I put on a ‘mask’ in order to display the emotions I need for the job.

6. I put on an act in order to deal with students or their parents in an appropriate way.

*Deep acting*

1. I try to actually experience the emotions that I must show to students.

2. I make an effort to actually feel the emotions that I need to display towards students.

3. I work hard to feel the emotions that I need to show to students.

4. I work at developing the feelings inside of me that I need to show to students.

*Expression of naturally felt emotions*

1. The emotions I show students match what I spontaneously feel.

2. The emotions I show students come naturally.

3. The emotions I express to students are genuine.

***The Faculty Teaching Efficacy scale (FTE)***

*Course design*

1. Have sufficient professional ability to teach the courses I am teaching.

2. Establish comprehensive teaching objectives.

3. Select appropriate teaching material.

4. Arrange appropriate timeline for the curricular progress.

5. Prepare my teaching material before class sessions.

*Instructional strategy*

1. Teach according to students’ various levels of readiness.

2. Utilize effective teaching methods to improve students’ grades.

3. Modify my teaching activities during class sessions in order to sustain students’ attention.

4. Have confidence in inspiring and maintaining students’ learning motivation.

5. Utilize various inquiring skills to stimulate students’ higher level thinking skills and discussions.

*Classroom management*

1. Promote a democratic environment in class.

2. Nurture a pleasant learning environment.

3. Maintain a good relationship with my students.

4. Share my personal experiences with students in order to promote emotional bonding.

5. Listen to my students in order to understand their thoughts.
